# Transgene *CpNAC68* from Wintersweet (*Chimonanthus praecox*) Improves Arabidopsis Survival of Multiple Abiotic Stresses

**DOI:** 10.3390/plants10071403

**Published:** 2021-07-09

**Authors:** Jie Lin, Daofeng Liu, Xia Wang, Sajjad Ahmed, Mingyang Li, Nik Kovinich, Shunzhao Sui

**Affiliations:** 1 Chongqing Engineering Research Center for Floriculture, Key Laboratory of Horticulture Science for Southern Mountainous Regions of Ministry of Education, College of Horticulture and Landscape Architecture, Southwest University, Chongqing 400715, China; lj0215@yorku.ca (J.L.); liu19830222@163.com (D.L.); wx221069@email.swu.edu.cn (X.W.); limy@swu.edu.cn (M.L.); 2 Department of Biology, Faculty of Science, York University, Toronto, ON M3J 1P3, Canada; ahmed80@yorku.ca

**Keywords:** *CpNAC68*, transcription factor, *Chimonanthus praecox*, abiotic stress, cold tolerance

## Abstract

The NAC (NAM, ATAFs, CUC) family of transcription factors (TFs) play a pivotal role in regulating all processes of the growth and development of plants, as well as responses to biotic and abiotic stresses. Yet, the functions of NACs from non-model plant species remains largely uncharacterized. Here, we characterized the stress-responsive effects of a NAC gene isolated from wintersweet, an ornamental woody plant that blooms in winter when temperatures are low. CpNAC68 is clustered in the NAM subfamily. Subcellular localization and transcriptional activity assays demonstrated a nuclear protein that has transcription activator activities. qRT-PCR analyses revealed that *CpNAC68* was ubiquitously expressed in old flowers and leaves. Additionally, the expression of *CpNAC68* is induced by disparate abiotic stresses and hormone treatments, including drought, heat, cold, salinity, GA, JA, and SA. Ectopic overexpression of *CpNAC68* in *Arabidopsis thaliana* enhanced the tolerance of transgenic plants to cold, heat, salinity, and osmotic stress, yet had no effect on growth and development. The survival rate and chlorophyll amounts following stress treatments were significantly higher than wild type Arabidopsis, and were accompanied by lower electrolyte leakage and malondialdehyde (MDA) amounts. In conclusion, our study demonstrates that *CpNAC68* can be used as a tool to enhance plant tolerance to multiple stresses, suggesting a role in abiotic stress tolerance in wintersweet.

## 1. Introduction

Plants have evolved complex responses to mitigate the effects of abiotic stresses. These include long-term physiological adaptations, such as changes in stature and lifecycle, and rapid molecular responses that alleviate the damaging effects of stress [1]. At the genetic level, rapid responses, including the activation of transcription factors (TFs) activate or repress the expression of genes encoding antioxidant enzymes [2], cell wall-related genes [3], and TFs [4]. NAC (NAM, ATAF1/2, CUC) is one of the largest TF families and is unique to plants. NAC proteins regulate genes by directly binding specific *cis*-acting DNA elements to activate or repress gene expression at the transcription level [5,6].

In the past decades, a growing number of NAC family genes have been identified and characterized. Most of them are widely involved in the regulation of various developmental, physiological, or stress-responsive processes. Compared with the typical helix-turn-helix structure of DNA binding, NAC TF proteins have unique domain characteristics [7]. All proteins in this family share a conserved N-terminal NAC domain, containing A, B, C, D, E subdomains. NAC proteins function as homo- or hetero-dimers, providing multiple functions due to varying compositions of monomers. The N-terminal NAC domain has an abundance of positive charges that are believed to enable the one side of the protein dimer to directly bind to DNA [8]. Nuclear localization signals in the C and D sub-region sequences may also be involved in binding *cis*-acting elements [7].

As one of the largest TF families in plants, NACs are divided into different subfamilies according to the sequence of their NAC domain. The subfamilies are NAP (NAC-like, Activated by AP3/PI), ATAF1/2 (*Arabidopsis thaliana* activation factor1/2), NAM (no apical meristem), and CUC (cup-shaped cotyledon). The NAP subfamily is reported to mainly regulate senescence, such as *AtNAP* [9], *OsNAP* [10], *CitNAC* [11], and *ApNAP* [12]. Functional characterization of these genes demonstrated that they are involved in leaf senescence [9,11,12,13]. The NAM subfamily mainly regulates the formation of apical meristems [14,15], whereas the CUC subfamily affects general growth and development [16]. The VND (vascular-related NAC-domain) subfamily may have a role in the development of secondary vascular tissues in woody plants [17,18,19,20], and specifically in mediating programmed cell death [19]. The ATAF subfamily regulates responses to biotic and abiotic stresses and can improve stress tolerance when expressed ectopically in non-host plant species [21]. For example, overexpressing *SgNAC1* and *SgNAC2* from *Stylosanthes guianensis* in tobacco enhanced tolerance to cold stress [22]. Moreover, ectopic expression of *HaNAC1* could augment drought stress tolerance in transgenic Arabidopsis [23].

NAC family genes are widely involved in plant growth [24], fiber development [25], lateral root development [26], fruit quality [27], ripeness of fruit [28], biotic [29] and abiotic stress responses [30], hormone responses [31], senescence [32,33], programmed cell death [34], and other physiological activities. Since the majority of NAC TFs regulate diverse processes, they are promising candidate genes in plant breeding [35]. However, research on NAC TFs has extensively focused on model plants such as *A. thaliana* [36], and select crop species, such as *Solanum lycopersicum* [37], *Populus* [38], *Oryza sativa* [39,40], *Triticum aestivum* [41], and *Zea mays* [42].

Wintersweet (*Chimonanthus praecox* (L.) Link) belongs to the Calycanthaceae family and is a perennial deciduous shrub. As a traditional and famous ornamental landscape plant in China, wintersweet blooms in cold winter with sweet fragrance [43]. Research on wintersweet has mainly focused on the molecular mechanisms of flower development [44], floral scent [45,46], flowering time [47], and the regulation of volatile compound [48] and flavonoid biosynthesis [49].

TFs may be involved in the regulation of different processes, including growth, flower development, metabolic pathways, and abiotic and biotic stresses [4]. In order to understand the biological functions of the NAC gene family in wintersweet, we isolated the cDNA of *CpNAC68* based on transcriptome data [50]. We assessed *CpNAC68* for the transcriptional activation activity, subcellular localization, and physiological functions ectopically in Arabidopsis under various abiotic stresses. The evidence shows that *CpNAC68* is a NAM-type NAC TF that confers enhanced tolerance to multiple abiotic stresses.

## 2. Results

### 2.1. Cloning and Sequence Analysis of CpNAC68

**Figure 1:**
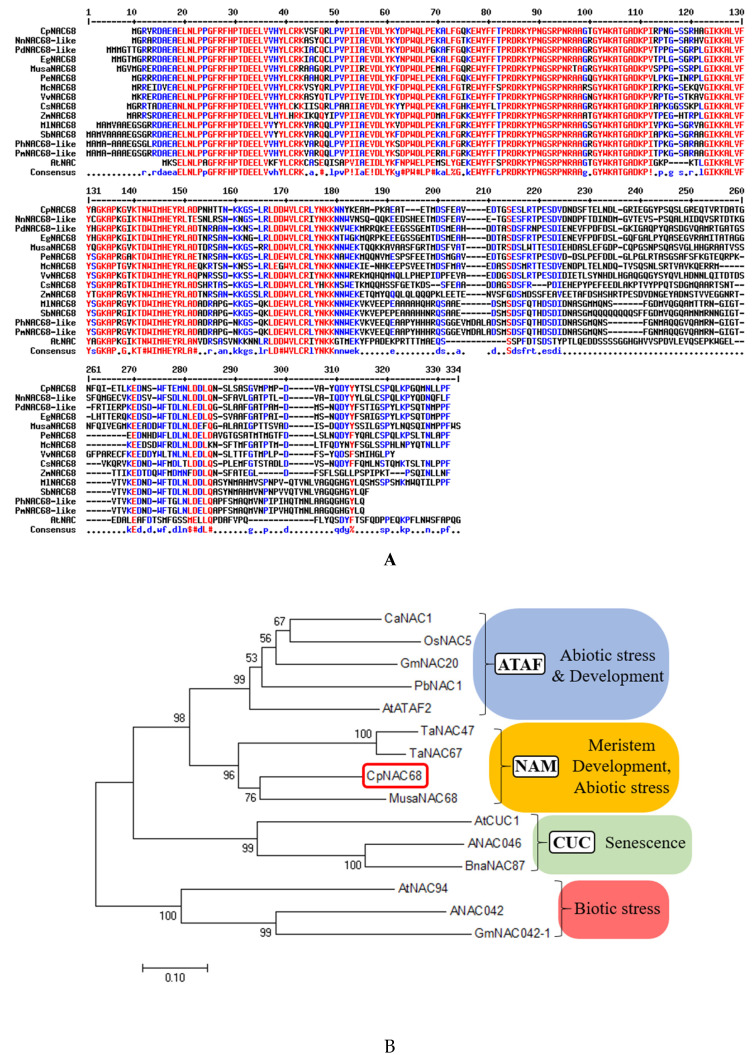
Sequence analysis of *CpNAC68*. (**A**) Multiple sequence alignment of CpNAC68 with other NAC68 TFs. The red sequence shows highly conserved subdomains. (**B**) Cluster analysis of CpNAC68 (indicated by a red frame) and NAC proteins from other species. Accession numbers: *Nelumbo nucifera* (XP_010257746.1), *Phoenix dactylifer* (XP_008782755.1), *Elaeis guineensis* (XP_010921285.1), *Musa acuminata* (XP_009399922.1), *Phalaenopsis equestris* (XP_020578930.1), *Macleaya cordata* (OVA03901.1), *Vitis vinifera* (XP_002283807.1), *Crocus sativus* (ABU40779.1), *Zostera marina* (KMZ65511.1), *Miscanthus lutarioriparius* (AIS74871.1), *Sorghum bicolor* (XP_002458677.1), *Panicum hallii* (XP_025816775.1), *Panicum miliaceum* (RLM92289.1), *A. thaliana* (NP_680161.1), *Capsicum annuum* (AY71222.1), *O. sativa* (AB028184.1), *Glycine max* (EU440353.1), *Phalaenopsis bellina* (KY979200.1), *A. thaliana* (AT5G08790), *T. aestivum* (KT345698.1), *T. aestivum* (KF646593.1), *A. thaliana* (AT3G15170), *A. thaliana* (AT3G04060), *Brassica napus* (KP641357.1), *Amborella trichopoda* (XP_006827219.2), *A. thaliana* (AT2G43000), *G. max* (KRH73619).

We cloned the largest open reading frame (ORF) of *CpNAC68* from the wintersweet leaf cDNA. Sequencing revealed that it was 906 bp, encoding a predicted protein of 302 amino acids (GenBank accession MZ501790). According to software predictions, the molecular weight of CpNAC68 is 34.32 kDa, and its theoretical isoelectric point (PI) and the instability index are 5.84 and 39.63, respectively. CpNAC68 lacks an obvious signal peptide or transmembrane domain sequence, as is observed in many other NAC proteins [51]. Secondary structure prediction indicated that it contains 25.91% α helix, 10.96% extended strands, 3.65% β turn, and 59.47% random coil. BlastP identified several characterized NAC68s from different plant species. Multiple sequence alignment showed that the N-terminal half of CpNAC68 has the conserved domain, including A, B, C, D, and E sub-domains [8], while the C-terminal half is highly divergent (Figure 1A). Sequence analysis revealed that *CpNAC68* is a member of the NAM subfamily and shares the highest amino acid sequence similarity with the *CmNAC68*-like protein from *Cinnamomum micranthum* (79.67%). A neighbor-joining (NJ) cluster tree with various characterized NACs clustered CpNAC68 with MusaNAC68, TaNAC47, and TaNAC67 (Figure 1B). All three of these genes have been identified to improve tolerance to multiple abiotic stresses.

### 2.2. Subcellular Localization and Transcriptional Activation Assay

**Figure 2:**
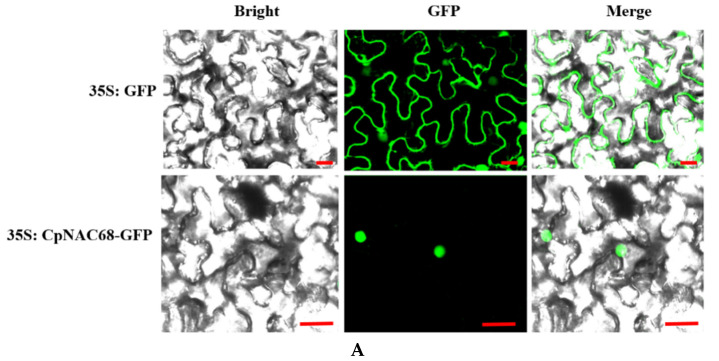

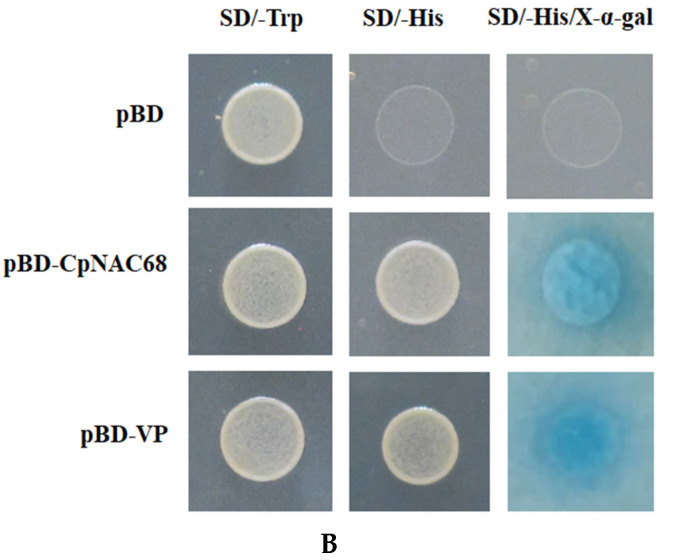
Subcellular localization and transcriptional activation activity of CpNAC68. (**A**) The control vector 35S:GFP and the recombinant vector 35S:CpNAC68-GFP were expressed in *N. benthamiana* leaf epidermal cells. Confocal images were captured 36 h after agro-infiltration and analyzed by laser scanning confocal microscope in bright, dark, and merged fields. Scale bar = 50 μm. (**B**) Y2H Gold yeast transformed with pGBKT7 (pBD) (negative control), pGBKT7−CpNAC68 (pBD-CpNAC68), and pGBKT7−VP (pBD-VP) (positive control). Growth and the β–galactosidase activity was assessed by SD/Trp-, SD/His- plates and in the presence of X-α-gal, respectively.

In order to determine the subcellular localization of CpNAC68, the plasmid *35S:CpNAC68-GFP* was constructed and *Agrobacterium tumefaciens* was used to infiltrate young leaves of *Nicotiana benthamiana* epidermal cells. An empty vector (35S:GFP) was used as a control. CpNAC68-GFP was only observed in punctate spherical structures reminiscent of nuclei, while the GFP signal was scattered throughout the cell periphery (Figure 2A). Since the spherical structures were only one per cell, these results strongly suggest that *CpNAC68* localizes to the nucleus, which is in accordance with the function of CpNAC68 as a TF.

To assess whether CpNAC68 has transcriptional activation activity, the yeast expression plasmid *pGBKT7-CpNAC68* was constructed by fusing the *CpNAC68* CDS with a GAL4 DNA-binding domain (DBD) in the pGBKT7 vector. The empty pGBKT7 and *pGBKT7-VP16* were used as the negative and positive controls, respectively. These three plasmids were transferred into the Y2H Gold yeast strain. The results demonstrate that the three transformants grew normally on SD/Trp plates, indicating that these three plasmids were introduced successfully. Transformants were then plated on the SD/His/X-α-gal. The yeasts carrying *pGBKT7-CpNAC68* or *pGBKT7-VP* grew and showed β-galactosidase activity (Figure 2B). The results indicate that CpNAC68 has activity as a transcriptional activator.

### 2.3. Expression Analysis of CpNAC68

**Figure 3:**
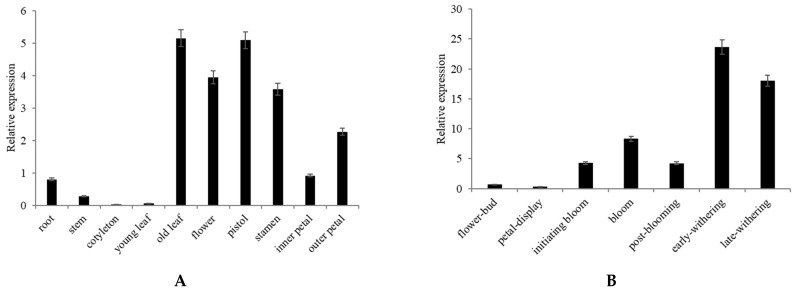

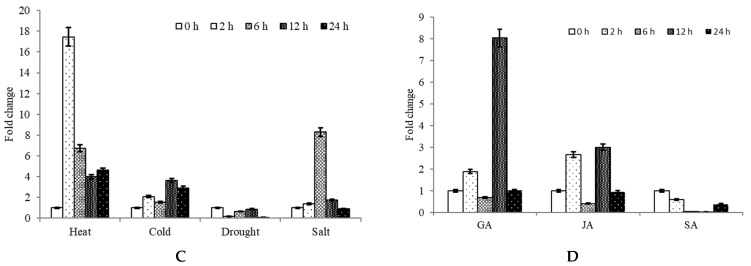
Analysis of *CpNAC68* expressions by qRT-PCR. (**A**) Expressions in different tissues and flora organs. (**B**) Flower development stages. (**C**) Stress treatments (heat, cold, drought, salt) at indicated time points. (**D**) Hormone treatments (GA, JA, SA) at indicated time points. Expression levels were normalized by *CpActin*. Data represent the mean of three biological repeats ± SD. Error bars indicate the standard deviation.

qRT-PCR found that *CpNAC68* is preferentially expressed in mature leaves and flowers (Figure 3A). It is also highly expressed in pistils compared to other floral organs (Figure 3A). There is a gradual rise in expression level with flower development (Figure 3B). To investigate the expression pattern of *CpNAC68* under various abiotic stresses and hormone treatments, RNA was extracted from stress-treated young leaves and the transcript levels were measured by qRT-PCR. *CpNAC68* was induced by heat, salt, and cold with different temporal dynamics (Figure 3C). Expressions peaked early (2 h) during heat, were delayed (6 h) during salt, and were late (12 h) during cold, respectively. By contrast, drought suppressed expressions. For the hormone treatments, *CpNAC68* was induced by GA and JA with different temporal dynamics, and expressions were suppressed by SA treatment (Figure 3D). Therefore, *CpNAC68* is positively and negatively regulated by specific stresses and hormones.

### 2.4. Abiotic Stress Tolerance of Transgenic Arabidopsis Overexpressing CpNAC68

**Figure 4:**
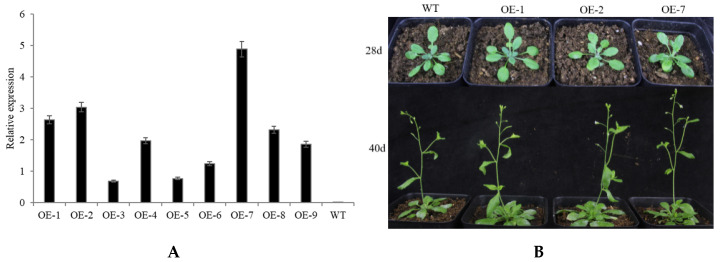
Phenotype observation of *CpNAC68* in transgenic Arabidopsis. (**A**). qRT-PCR identification of *CpNAC68* transcript levels in the leaves of nine overexpressed Arabidopsis lines and WT plants. *AtActin* was used as the internal reference gene; bars indicate the SE of the mean from three technical replicates and three biological replicates. (**B**) Phenotype observation of different transgenic lines and WT plants were at different developmental stages. Twelve individual plants for each line were used for height measurements (Figure S1). Bars correspond to standard error.

To further investigate the function of *CpNAC68*, an overexpression vector pGWB551-*CpNAC68* was constructed and transferred into Arabidopsis. Following the selection of the T1 seeds, homozygous T2 lines were selected on hygromycin and those with the highest expression of *CpNAC68* were identified by qRT-PCR (Figure 4A). We used the T3 lines that had the highest expression levels (namely, OE1, OE2, OE7) for further functional analysis. In the absence of stress treatments, no obvious changes in growth phenotype were observed (Figure 4B).

**Figure 5:**
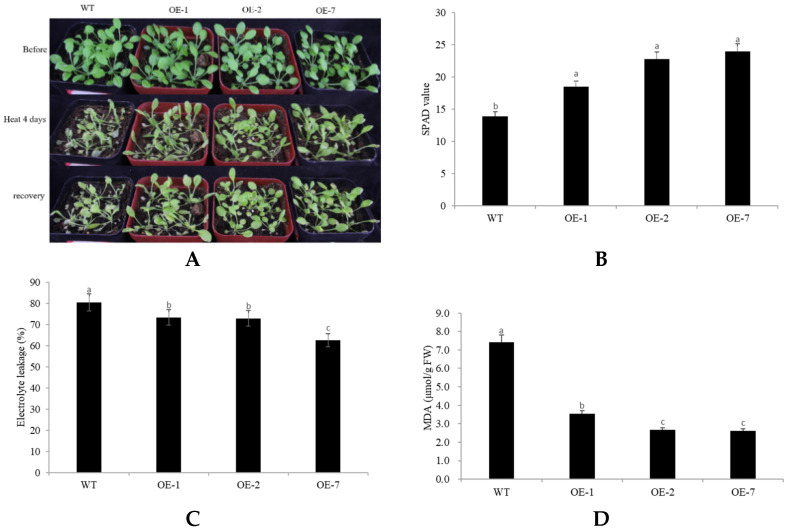
Ectopic expression of *CpNAC68* confers enhanced tolerance to heat stress in transgenic Arabidopsis. (**A**) Performance of overexpression in OE lines and WT plants after heat stress for 4 days, followed by recovery at normal conditions for 3 days. Images were taken at the indicated time points. (**B**) SPAD values of OE lines and WT plants under heat treatment. (**C**) Electrolyte leakage of plants under heat treatment. (**D**) MDA content of plants under heat treatment. Values are the averages of three independent repeated trials. Data represent the mean ± (SE). Bars correspond to the standard error. The significance test was performed by Duncan’s multiple range test, which is indicated by different letters.

In order to ascertain the response of *CpNAC68* overexpression to heat stress, plants were subjected to a high temperature (42 °C) for 3 days. The transgenic lines clearly displayed less photobleaching, etiolation, and wilting, whereas the WT plants exhibited large areas of chlorosis and stagnation (Figure 5A). As another indicator of enhanced stress tolerance, the transgenic lines OE1, OE2, and OE7 exhibited soil plant analysis development (SPAD) values of 18.45, 22.74, and 23.96, respectively, which were significantly higher than that of the WT plants (13.87). This indicates that *CpNAC68* alleviates damage judged by the higher chlorophyll amounts in transgenic lines (Figure 5B). In support of this, the electrolyte leakage was 73.27%, 72.82%, and 62.61%, respectively, which was significantly lower than that of the wild type (80.31%) (Figure 5C). Moreover, the molar concentration of MDA, an indicator of lipid peroxidation degree, varied from 2.61 to 3.54 μmol/g FW, which was less than half of that of the WT plants (7.43 μmol/g FW) (Figure 5D). These results demonstrate that the overexpression of *CpNAC68* enhanced the heat tolerance of transgenic plants.

**Figure 6:**
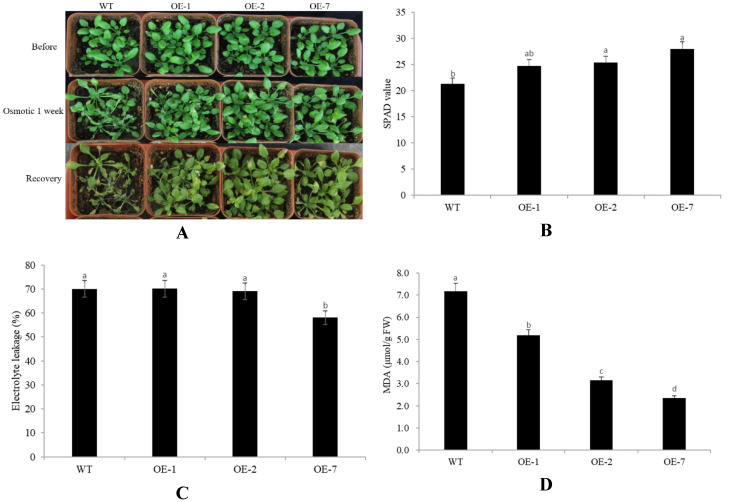
Ectopic expression of *CpNAC68* confers improved tolerance to osmotic stress in transgenic Arabidopsis. (**A**) Performance of OE lines and WT plants after osmotic stress for 1 week, followed by recovery at normal conditions for 3 days. Images were taken at the indicated time points. (**B**) SPAD values of OE lines and WT plants under osmotic treatment. (**C**) Electrolyte leakage of OE lines and WT plants under osmotic treatment. (**D**) MDA content of OE lines and WT plants under osmotic treatment. Data represent the averages of three independent repeated trials. Values represent the mean ± SE. Bars correspond to the standard error. The significance test was performed by Duncan’s multiple range test, which is indicated by different letters.

To explore the role of *CpNAC68* as a regulator of osmotic stress in plants, both the OE lines and WT plants were irrigated with 20% PEG. After one week of treatment, we observed that the WT plants were dehydrated and wilting. On the contrary, the transgenic Arabidopsis remained green and healthy. After 10 days of the treatment, most of the WT Arabidopsis died (Figure 6A).

The SPAD values of the *CpNAC68* transgenic lines were 24.74, 25.37, and 27.99, which were significantly higher than that of the WT (21.35) (Figure 6B). No differences in electrolyte leakage were observed among the OE-1, OE-2, and WT plants, whereas the electrolyte leakage of OE-7 was significantly lower than the WT plants (58.04% compared to 70.02%, respectively) (Figure 6C). The MDA levels varied from 2.35 to 5.91 μmol/g FW, which was markedly lower than the WT plants (7.81 μMOL/g FW) (Figure 6D). Thus, our data show that the overexpression of *CpNAC68* confers improved osmotic tolerance in the transgenic Arabidopsis.

**Figure 7:**
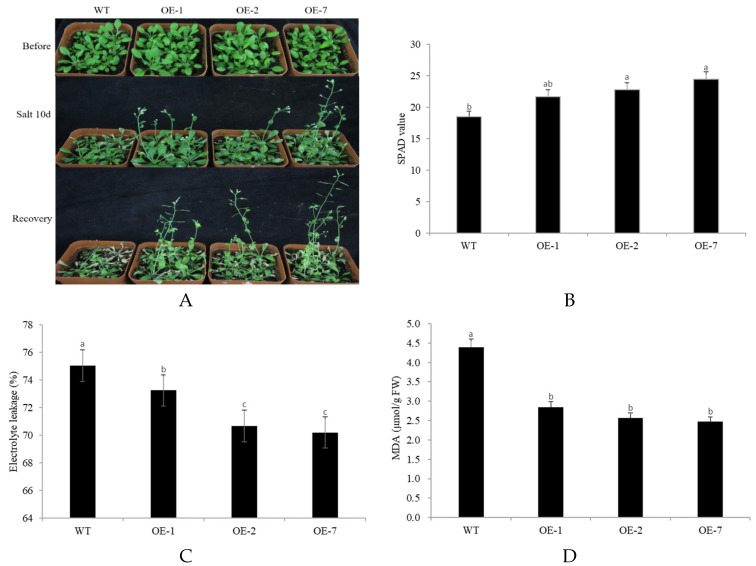
Overexpression of CpNAC68 confers enhanced tolerance to salt stress in transgenic Arabidopsis. (**A**) Performance of OE lines and WT plants after salt stress for 10 days, followed by recovery at normal conditions for 4 days. Images were taken at the indicated time points. (**B**) SPAD value of OE lines and WT plants under salt treatment. (**C**) Electrolyte leakage of OE lines and WT plants under salt treatment. (**D**) MDA content of OE lines and WT plants under salt treatment. Values represent the averages of three independent repeated trials. Data represent the mean ± SE. Bars correspond to the standard error. The significance test was performed by Duncan’s multiple range test, which is indicated by different letters.

To assess for salt stress tolerance, 500 mL of NaCl solution with a concentration of 200 mMOL was poured into the watering trays every 5 days. After one week, the transgenic lines bloomed and produced pods normally, while the WT plants showed almost no growth even after two weeks (Figure 7A). The SPAD values of OE-1, OE-2, and OE-7 were 21.67, 22.75, and 24.44, respectively, which were significantly higher than that of the WT plants (18.49) (Figure 7B). The electrolyte leakage was 73.26% for OE-1, 70.68% for OE-2, and 70.21% for OE-7, which was significantly lower than that of the WT plants (75.05%) (Figure 7C). The MDA levels were about 2.6 μMOL/g FW, which was almost half of that of the WT (4.39 μMOL/g FW) (Figure 7D). In summary, under high salt stress, the membrane system of transgenic *CpNAC68* Arabidopsis is more resistant to damage than the WT.

**Figure 8:**
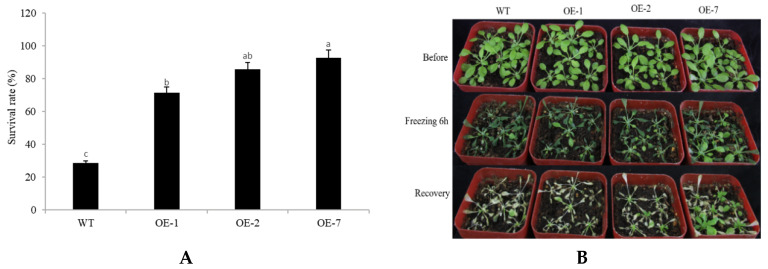
Improvement of freezing tolerance using *CpNAC68*-OE in Arabidopsis. (**A**) Survival rates of *CpNAC68* OE and WT plants under freezing stress. Values represent the mean ± SE (*n* = 3). Bars correspond to the standard error. The significance test was performed by Duncan’s multiple range test, which is indicated by different letters. (**B**) *CpNAC68* OE and WT seedling performance under freezing stress. One-month-old plants were subjected to −4 °C for 6 h, then transferred back to normal conditions to promote recovery for 3 days. Pictures were taken after each time point.

Finally, we assessed cold stress tolerance. After 6 h of low temperature (−4 °C) treatment, the transgenic lines exhibited impressively higher survival rates at 71.42%, 85.71%, and 92.85%, compared to the WT (28.57%) (Figure 8A). After being returned to a normal temperature for 3 days, most of the transgenic plants resumed growth, while the WT plants remained wilting and eventually died (Figure 8B). Thus, *CpNAC68* participates in cold stress tolerance when overexpressed in Arabidopsis.

## 3. Discussion

Over the last decade, genes of the NAC family have been characterized mainly by model plant species. Notable studies have shown the diversity of the NAC family and the complexity of their functions, including complex miRNA regulatory mechanisms at the level of transcription [52], and NAC protein phosphorylation and ubiquitination [53]. In this study, we isolated the *CpNAC68* gene from wintersweet. Protein sequence analysis revealed that the N-terminal half of CpNAC68 possesses a conserved domain of the NAC gene family, specifically NAM subfamily-like domain. Bioinformatics analysis indicated that the CpNAC68 protein is hydrophilic and contains no obvious signal peptide or transmembrane domain, like most other NACs [51]. Clustering CpNAC68 with NAC proteins demonstrated the closest amino acid similarity to the drought and salt tolerance protein MusaNAC68 from *M. Acuminate*, and multiple abiotic tolerance proteins TaNAC47 and TaNAC67 from *T. aestivum*. Since *CpNAC68* expression was found to be upregulated during wintersweet flowering, which is stimulated by cold temperatures, and the protein was similar to *MusaNAC68*, *TaNAC47*, and *TaNAC67*, we hypothesized that *CpNAC68* has a role in regulating abiotic stress tolerance.

Localization of the CpNAC68-GFP fusion protein in *N. benthamiana* cells found fluorescent signals only in the nucleus. This is similar to most characterized NACs, such as SNAC3 [54], AaNAC1 [55], GhNAC2 [56], and CaNAC064 [57]. However, some involved in regulating pathogen stress responses, such as GFP-GmNAC42-1 from soybean, have also been detected in the cytosol [58]. Consistent with a role as a TF, CpNAC68 had transcriptional activation activity when expressed in yeast. Previous studies have reported that a large number of NAC TFs have transcriptional activation functions, such as ONAC063 [59], OsNAC111 [60], CaNAC064 [57], ChNAC1 [61], and GmNAC42-1 [58].

*CpNAC68* was expressed in all of the wintersweet tissues tested, with the highest expression in old leaves and flowers (Figure 3A). In addition, *CpNAC68* had different expression levels at different flower development stages, with the highest expressions observed during the bloom stage (Figure 3B). Thus, *CpNAC68* may regulate cellular or physiological factors to provide stress tolerance during wintersweet flower senescence. *CpNAC68* was upregulated by cold, heat, and salt stresses (Figure 3C). While *CpNAC68* demonstrated some considerable fluctuations in gene expression in response to GA and JA (Figure 3D), similar to *LrNAC35* [62] and *OoNAC72* [63], some consistent changes in gene expression were observed in response to other hormones and stress treatments. However, *CpNAC68* was downregulated by drought and SA treatment (Figure 3C,D), which is in contrast with *MusaNAC68* [64]. Other NACs, such as *SNAC3* were induced by drought, salt, heat, oxidative stress, and ABA, but suppressed by cold, submergence, and wounding [54]. *GmNAC081* and *GmNAC030* were implicated in triggering leaf senescence, and were induced by drought, salt, and cold, as well as ABA, SA, MeJA [65]. Thus, we speculated that *CpNAC68* may be involved in regulating heat, cold, salt, and other stresses.

To validate our hypothesis and better understand the function of *CpNAC68*, we overexpressed its largest ORF in Arabidopsis. We found no obvious phenotypic differences between the transgenic lines and the WT plants. This is notable since most ectopically overexpressed NAC genes will not adversely affect the development of transgenic plants [56,66]. They can also cause curly and shrunken leaves and dwarfing [17]. Under the heat, osmotic, and salt stresses, the survival rate and chlorophyll SPAD values of transgenic *CpNAC68* Arabidopsis were significantly higher than that of the WT plants, while the relative electrolyte leakage and the MDA content were significantly lower than that of the WT plants. Collectively, our results indicate that the overexpression of *CpNAC68* confers enhanced tolerance to multiple stresses and thus, has a positive regulatory role in stress response processes in Arabidopsis.

Currently, there has been one other NAC68 protein that was functionally characterized. *MusaNAC68* conferred enhanced tolerance to drought and high salt stress when expressed in transgenic *M. acuminata* [64], and it reduced xylem secondary wall thickness [67]. Differently than *MusaNAC68, CpNAC68* was downregulated by drought and confers tolerance to other stresses (i.e., cold, heat, osmotic, and salt) when ectopically expressed in Arabidopsis. Some other NACs have been shown to confer tolerance to multiple stresses. Transgenic *SNAC3* rice grew better than the WT plants under high temperatures, drought, and oxidative stress [54]. Overexpressing *ONAC063* in Arabidopsis could confer improved tolerance to salt, heat, and osmotic stress [59]. Furthermore, with involvement in the ABA-dependent signaling pathway, *SlNAC1* rendered the enhanced resistance of transgenic plants under drought, salt, and cold stress [68], and *TaNAC47* [66] and *TaNAC67* [69] simultaneously enhanced these three abiotic stress tolerances. However, some NAC genes reduce stress tolerance. For example, the ectopic overexpression of *BoNAC019* in Arabidopsis reduced survival rate, proline, and ABA content, under drought stress. Similarly, *LpNAC13* reduced drought tolerance in transgenic tobacco, but improved salt tolerance, despite being induced by drought, salt, cold, and ABA treatments [70]. Overall, various NAC genes play multiple complex roles in regulating abiotic stress tolerance in different species. Future work needs to concentrate on the mechanism of action of *CpNAC68* on downstream genes and metabolites in wintersweet.

## 4. Materials and Methods

### 4.1. Plant Materials and Growth Conditions

For gene expression profiling, seeds of wintersweet were cultivated in a greenhouse at Southwest University, Chongqing, China. Tissues, including the root, stem, cotyledon, leaves, flowers, and floral organs in full bloom (stamen, pistil, outer petal, middle petal, and inner petal), were dissected and flash-frozen in liquid nitrogen. According to the classification of wintersweet floral stages [44], the flowers were collected at the lower-bud, petal-display, initiating bloom, bloom, post-blooming, early-withering, and late-withering stages, respectively.

*N. Benthamiana* was used for the subcellular localization assay. *N. benthamiana* seedlings were grown under a 16 h light (2000 lux) and 8 h dark photoperiod with a constant temperature (22 ± 1 °C) and relative humidity (75% RH).

For plant transformation, seeds of Arabidopsis ecotype Columbia−0 (Col−0) were vernalized under 4 °C for three days, then sterilized in 2% sodium hypochlorite solution, followed by five rinses with sterile water. Subsequently, the sterilized seeds were spread on MS plates. The MS plates containing the Arabidopsis seeds were stored in a plant incubator with the same photoperiod as *N. benthamiana* (22 ± 1 °C, 75% RH). After two weeks, the seedlings were transplanted into a 1:1 mixture of peat and vermiculite and grown under the same conditions as above.

### 4.2. Cloning and Sequence Analysis

Total RNA was isolated from the plant tissues (lyophilized powder) using the Plant RNA Purification Kit (Tiangen Biotech, Beijing, China) following the manufacturer’s instructions. For quality and quantity control, the RNA extracts were visualized by 1% agarose gel electrophoresis, then quantified with a Nano-Drop ND-1000 spectrophotometer (Thermo Fisher Scientific, Wilmington, MA, USA) at optical densities of 260 and 280 nM.

The first-strand cDNA was synthesized from total RNA using a PrimeScript RT Reagent Kit with gDNA Eraser (TaKaRa, Otsu, Japan). *CpNAC68* was amplified from cDNA templates (mixed with leaves and flower tissues) accompanied with the specific primers *CpNAC68*-F/R (Table S1). Subsequently, the products above were isolated and confirmed by sequencing, as described by Huang et al. [47]. The sequence identity of *CpNAC68* was determined by a homology search in the National Center for Biotechnology Information (NCBI) database by the Protein-BLAST program (http://www.ncbi.nlm.nih.gov/, accessed on 3 May 2019). The amino acid sequences of various NAC proteins from other species for multiple sequence alignment and cluster tree construction were retrieved from the NCBI database. Multiple sequence alignment was performed using the online tool Multalin (http://multalin.toulouse.inra.fr/multalin/, accessed on 5 July 2020). The cluster analysis tree was generated by MEGA 7.0 with the neighbor-joining (NJ) method [71].

### 4.3. CpNAC68 Expression Analyses

The expression profiles of *CpNAC68* of different tissues, organs, and floral stages, as well as stress and chemical treatments, were determined by qRT-PCR analyses with the primers *CpNAC68*-qF/qR (Table S1). qRT-PCR was performed as described by Liu et al. [44]. *CpActin* and *AtActin* were used as reference genes to normalize the data. The relative gene expression levels were calculated by the Ct method (2^–ΔΔCt^) [72]. Each reaction comprised three biological and technical replicates [73].

### 4.4. Subcellular Localization and Transactivation Activity Assay of CpNAC68

Specific primers *CpNAC68*-slF/slR (Table S1), containing *KpnⅠ* and *XbaⅠ* restriction sites, were designed to clone *CpNAC68* without a termination codon (TGA) into pCAMBIA1300. The *pCAMBIA1300::CpNAC68-GFP* and the empty vector were separately infiltrated into *N. benthamiana* epidermal cells mediated by *A. tumefaciens* strain GV3101. The GFP fluorescence was observed by confocal laser microscopy (Olympus, Japan).

pGBKT7 was digested with *NdeⅠ* and *NotⅠ*; the related primers are listed in Table S1. Then, the recombinant plasmid was transformed into the Y2H Gold yeast strain. pGBKT7-VP and pGBKT7 were utilized as the positive and negative controls, respectively. We used SD/Trp plates to select positive transformants. For the transactivation assay, SD/His/X−α-gal plates were used to evaluate the transcriptional activity.

### 4.5. Overexpressed Plasmid Construction and Arabidopsis Transformation

*CpNAC68* was fused in-frame into the pGWB551 vector by the Gateway recombination reactions with specified primer *CpNAC68*-gF/gR, as shown in Table S1. After being confirmed by sequencing, the *pGWB551–CpNAC68* plasmid was transformed into *A. tumefaciens* to further explore the function of *CpNAC68*. Arabidopsis ecotype Col-0 was used for the genetic transformation, which was carried out by the floral dip method [74]. T0 seeds were sowed in MS media containing 25 mg/L hygromycin for transgene selection. The overexpression status of *CpNAC68* in the T3 homozygous line was confirmed by qRT-PCR under the normalization of *AtActin* with the primers listed in Table S1. Then, the three T3 homozygous lines with relatively higher expression levels were used for the phenotypic analyses in the stress tolerance experiments.

### 4.6. Stress Treatments and Phenotype Observation

In order to understand the gene expression patterns under different abiotic stresses and hormone treatments, three-month-old wintersweet seedlings were used. Heat treatment was conducted by transferring the seedlings to a growth chamber at 42 °C with a 16 h light/8 h dark cycle (2000 lux). Cold treatment was conducted by transferring the seedlings to a growth chamber at 4 °C with a 16 h light/8 h dark cycle (2000 lux). For the salt and drought stress treatments, the seedlings were grown in soil irrigated with 150 mM *NaCl* or 20% PEG 6000 (polyethylene glycol 6000), respectively. For exogenous hormone treatments, the wintersweet seedlings were treated with 10 µM GA, 100 µM JA, and 2 mM SA for 24 h, respectively. Two leaves of one individual wintersweet seedling were collected as one replicate at 0, 2, 6, 12, and 24 h after treatment, and frozen in liquid nitrogen. There were three biological replicates for each treatment.

To evaluate the drought, salt, heat, and freezing tolerance of the transgenic *CpNAC68* Arabidopsis, 4-week-old T3 plants were used. For heat treatment, the seedlings were exposed to 42 °C for 6 days, then returned to the normal conditions in a growth chamber. For freezing treatment, the seedlings were exposed to −4 °C for 4 hours, and subsequently returned to the normal growth conditions. For osmotic treatment, the seedlings were well irrigated for 4 weeks, then watered with 20% PEG 6000. For salt stress treatment, the seedlings were watered with a concentration of 200 mM NaCl solution every 5 days. The controlled plants were grown at 25 °C and were mock-treated with water. For each tested tissue, three biological replicates were collected by harvesting samples from three different plants.

The physiological parameters of stress tolerance were measured by the survival rate, the chlorophyll SPAD value, electrolyte leakage, and the MDA content. For freezing stress, the survival rate was scored one week after treatment. The chlorophyll SPAD values were determined according to Mao et al. [69]. Electrolyte leakage was measured as described [75]. The MDA content was measured using the thiobarbituric acid-based (TBA) method, following Robert L. Heath [76].

### 4.7. Statistical Analysis

SPSS (IBM SPSS Statistics 22) software was used to analyze the differences of paired data with Duncan’s multiple range tests. The values of *p* < 0.05 and *p* < 0.01 were indicative of statistical significance, which were recognized as statistically significant and extremely significant, respectively. All trials were independently repeated at least three times.

## 5. Conclusions

We cloned the NAM-subfamily NAC TF gene *CpNAC68* from wintersweet and provided evidence that it is a nuclear-localized activator of transcription. *CpNAC68* expressions were induced by multiple abiotic stresses (drought, salt, cold, and heat), as well as hormone treatments (SA, JA, and GA). Overexpressing *CpNAC68* ectopically in Arabidopsis enhanced tolerance to cold, heat, osmotic, and salt stresses. Thus, *CpNAC68* has a role in positively regulating stress tolerance.

## Data Availability

Not Applicable.

## Supplementary Materials

The following are available online at https://www.mdpi.com/article/10.3390/plants10071403/s1. Figure S1: Plant height of 40-day-old WT and transgenic plants; Table S1: List of primers used in this study.
