# Supplementary material for: Transgene CpNAC68 from Wintersweet (Chimonanthus praecox) Improves Arabidopsis Survival of Multiple Abiotic Stresses
Source: Plants (Basel). 2021 Jul 9;10(7):1403. doi: 10.3390/plants10071403 (PMC8309309; doi:10.3390/plants10071403)
Supplement: Supplementary file 1 [file plants-10-01403-s001.zip › plants-1271894-supplementary.pdf]

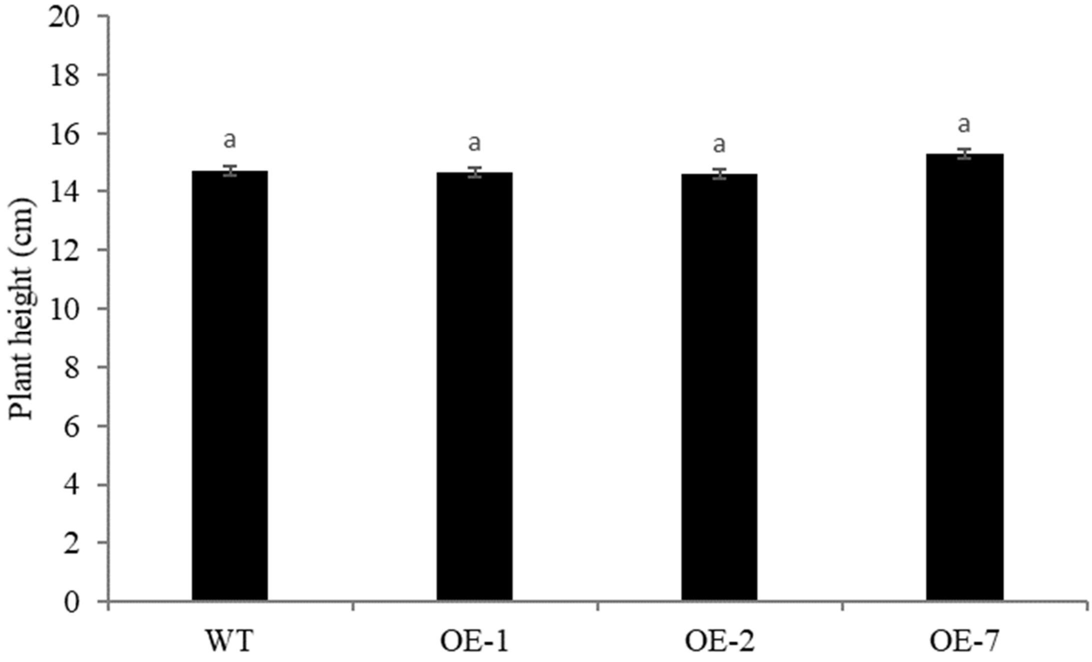

Figure S1. Phenotype observation of *CpNAC68* in transgenic Arabidopsis. Plant height of 40-day-old WT and transgenic plants. Data represent the mean ± (SE). The significance test was performed by Duncan's multiple range test, and the results of the groups are indicated by the letter at the top of the bars.

Table S1. List of primers used in this study.

Primer Name	Primer Sequence (5'→3')	Purpose
CpNAC68-F	AGAGATCAGCAGCATGGGGAG	cloning of *CpNAC68 cDNA*
CpNAC68-R	GGCATGGCAAGCATACTTGG	cloning of *CpNAC68 cDNA*
CpNAC68-qF	AGGCTTCAGCTGTGGAGAAC	qRT-PCR
CpNAC68-qR	TAGGCAGGGAGCAAACAGTG	qRT-PCR
CpActin-F	GTTATGGTTGGGATGGGACAGAAAG	qRT-PCR
CpActin-R	GGGCTTCAGTAAGGAAACAGGA	qRT-PCR
AtActin-F	CTTAGGTCTCGGTCGCAG	qRT-PCR
AtActin-R	ATCGGTGGTTAGGGACAC	qRT-PCR
CpNAC68-slF	cggggtaccACTCTCCCAAGATGGATGTGGTG	subcellular localization
CpNAC68-slR	gctctagaAAACGGTAGCAGGTTCATCTGAC	subcellular localization
CpNAC68-yF	ggaattccatatgAGAGATCAGCAGCATGGGGAG	transactivation activity
CpNAC68-yR	ataagaatgcggccgcGGCATGGCAAGCATACTTGG	transactivation activity
CpNAC68-gF	GGGGACAAGTTTGTACAAAAAAGCAGGCTAGAGATCAGCAG CATGGGGAG	expression vector construction
CpNAC68-gR	GGGGACCACTTTGTACAAGAAAGCTGGGTGGCATGGCAAGC ATACTTGG	expression vector construction
